# 3D Electron Microscopy Gives a Clue: Maize Zein Bodies Bud From Central Areas of ER Sheets

**DOI:** 10.3389/fpls.2020.00809

**Published:** 2020-06-11

**Authors:** Elsa Arcalís, Ulrike Hörmann-Dietrich, Lukas Zeh, Eva Stoger

**Affiliations:** Department of Applied Genetics and Cell Biology, University of Natural Resources and Life Sciences, Vienna, Vienna, Austria

**Keywords:** electron microscopy, volume electron microscopy, endomembrane system, endoplasmic reticulum, protein bodies, cereal endosperm, maize

## Abstract

Zeins are the main storage proteins in maize seed endosperm, and the onset of zein synthesis in young seeds challenges the endomembrane system and results in the formation of storage organelles. Even though zeins lack a conventional endoplasmic reticulum (ER) retention signal, they accumulate within the ER and assemble in conspicuous ER-derived protein bodies (PBs) stabilized by disulfide bridge formation and hydrophobic interaction between zein chains. Zein body formation during seed development has been extensively studied, as well as the mechanisms that lead to the initiation of PBs. However, the exact course of the PB formation process and the spatial relationship with the ER remain unclear. The development of serial block face scanning electron microscopy (SBF-SEM) techniques that allow three-dimensional imaging combined with the high resolution of electron microscopy provides new perspectives on the study of the plant endomembrane system. Here, we demonstrate that (i) the ER of maize seeds is mainly formed by massive sheets and (ii) PBs are not budding from tubules or the edge of sheets, but protrude from the entire surface of the ER sheet.

## Introduction

Cereal seeds are characterized by a high degree of functional specialization for the storage of proteins and energy. For example in maize, 70% of the seed proteome consists of storage proteins (Flint-Garcia et al., 2009). The main storage proteins are zeins, which are divided into four subfamilies termed α-, β-, γ-, and δ-zein. Zein synthesis in the endosperm starts as soon as 10 days after pollination (daps), increases steadily along development and reaches a peak around 25 daps (Woo et al., 2001; Arcalis et al., 2010). To support such a level of protein synthesis, the endoplasmic reticulum (ER) of the endosperm has to be highly active and well developed. Moreover, it needs to be flexible enough to be able to rapidly accommodate the synthesized proteins in newly formed storage organelles (Arcalis et al., 2014). Zeins do not carry a conventional ER retention sequence, but they assemble into aggregates within the ER lumen leading to the formation of protein bodies (PBs), surrounded by a ribosome studded membrane (Lending and Larkins, 1989). The induction of PBs has been extensively studied in maize (Lending and Larkins, 1989; Guo et al., 2013), but also in heterologous systems such as tobacco seeds, in which recombinant zeins from different subfamilies have been expressed (Coleman et al., 1996, 2004; Bagga et al., 1997).

In spite of the abundant literature about PBs, the exact mechanism of their formation is not yet fully understood, and little is known about the morphology and development of the ER in cereal endosperm. Most of the available data are based either on confocal or transmission electron microscopy (TEM). Confocal microscopy is an excellent tool that allows *in vivo* imaging and provides three-dimensional (3D) information. Moreover, the use of fluorescent tags in combination with different γ-zein fragments in leaves has revealed the crucial role of the N-terminal part of 27-kD γ-zein in the initiation of the formation of PBs (Llop-Tous et al., 2010), which is not restricted to specific tissues or organelles (Hofbauer et al., 2014). However, confocal microscopy has a limited resolution. Electron microscopy, on the other hand, offers a high resolution and early studies on PB biogenesis and distribution of zeins within the PBs were mostly based on TEM pictures providing detailed, but only two-dimensional information, at the ultrastructural level (Lending and Larkins, 1989). Modern 3D imaging techniques with nanoscale resolution include serial section TEM or electron tomography. Both techniques offer the possibility to image volumes at ultrastructural level, but the former is technically challenging and the latter is restricted to small volumes (Kittelmann, 2018). The recent development of block face imaging techniques such as serial block face scanning electron microscopy (SBF-SEM) facilitates the 3D study of ultrastructural features by generating large numbers of images aligned in the *z*-axis that allow not only the generation of 3D models but also the rapid screening of a large sample volume (Kittelmann, 2018).

In order to investigate the ultrastructure of the ER and its spatial relationship with nascent PBs and oil bodies in a highly specialized storage tissue, we have generated 3D models based on SBF-SEM of maize endosperm cells at two stages of seed development.

## Materials and Methods

### Plant Material

Experiments were performed in wild-type (WT) maize plants (HiII) grown in soil in a growth chamber with a 13-h photoperiod, 25/22°C day/night temperatures and 70% relative humidity. Seeds were harvested at two different developmental stages, 14 and 21 days after pollination (dap) that correspond to the stages 2 and 3 of maize seed development described in Arcalis et al. (2010).

### Sample Preparation for Electron Microscopy

Thin slices of maize seed tissue were cut under the silk hair scar with a razor blade, small tissue pieces of about 1 mm^3^ including the aleurone layer were then excised and immediately immersed in fixative solution (2.5% glutaraldehyde and 2% paraformaldehyde in 0.1 M cacodylate buffer, pH 7.4). After several washing steps with cacodylate buffer (0.1 M, pH 7.4), samples were subjected to osmium impregnation by incubation in 2% osmium tetroxide and 0.2% ruthenium red in 0.1 M cacodylate buffer (pH 7.4) for 1 h, followed by 1% (w/v) thiocarbohydrazide (TCH) solution in ultrapure water for 45 min and 2% aqueous osmium tetroxide for an additional hour. TCH acts as a mordant that allows deposition of additional osmium to the original osmium sites. Moreover, it makes the specimen more conductive to electrons, preventing excess surface charging of the sample that leads to poor quality images under the SEM (Tapia et al., 2012 and references therein). In order to improve the contrast of the sample, uranyl acetate (UA) was used in combination with lead aspartate. *En bloc* staining was performed overnight with 2% aqueous UA, followed by Walton’s lead aspartate (Walton, 1979) for 30 min. Tissue pieces were thoroughly rinsed after each step, before proceeding to the next. Samples were dehydrated through an ethanol series and prior to resin infiltration, samples were incubated in pure acetone. Dehydrated samples were progressively infiltrated in epoxy resin, embedded in flat molds and polymerized at 60°C during 24 h.

Resin blocks were trimmed in such a way that the aleurone layer was retained in all sections to facilitate orientation within the tissue. Sections with silver interferences were collected on copper grids and observed using a FEI Tecnai G2 transmission electron microscope operating at 160 kV. At least three sections per developmental stage were analyzed.

### Serial Block Face Imaging

Tissue blocks with an approximate surface of 3600 μm^2^ were fixed with silver conductive epoxy (TED PELLA Inc., United States) on 8–mm SBEM stubs (TED Pella Inc., United States) and hardened for 24 h at 60°C. Subsequently, samples were sputter-coated with a 40–nm gold layer prior to volume imaging in a Volumescope SEM (Thermo Fisher Scientific, United States). Images were collected at 1.78 kV, with a beam current of 100 pA. Pixel size was 5 nm and section thickness 40 nm. The obtained stack of images was scaled to 25% and aligned with ImageJ and the reconstruction of endomembranes was done with Amira software (Thermo Fisher Scientific, United States). At least five different stacks were obtained for each of the time points studied and one representative model of each developmental stage was generated.

## Results

**FIGURE 1:**
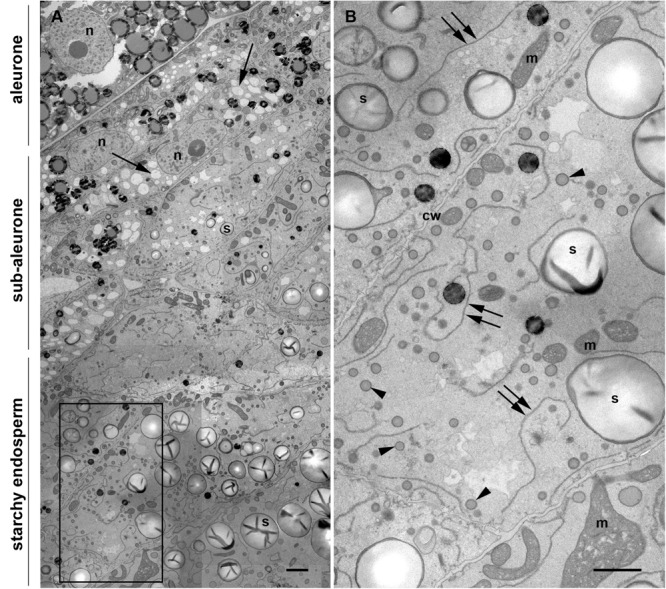
Transmission electron microscopy of a maize seed at mid-developmental stage (14 dap, Stage 2). **(A)** Composed image comprising aleurone, sub-aleurone, and starchy endosperm. The nucleus (n) and the prominent electron transparent vacuole-like structures (arrows), as well as small, scarce starch grains (s) are indicated in the sub-aleurone cells. Large and abundant starch granules (s) are found in the starchy endosperm. **(B)** Enlargement from inset in [(**A**), framed box], showing starchy endosperm cells with prominent ER strands (double arrows), abundant protein bodies (arrowheads) and starch grains (s). Cell walls (cw), mitochondria (m). Scale bars, 2 μm.

Once differentiated, the cereal endosperm consists of four different cell types, including the embryo-surrounding region, transfer cells, the starchy endosperm and the aleurone layer. The aleurone layer covers the entire perimeter of the endosperm except for the transfer cell region (Olsen, 2001). In the case of maize, the aleurone is a monolayer of regular, cuboidal cells, with a thick cell wall and a prominent nucleus (Figure 1A). Early in endosperm development, complete cellularization occurs by centripetal growth of the cell layers, toward the center of the endospermal cavity (Olsen, 2001). Subsequently, the final population of endosperm cells is generated via mitotic cell divisions, whereby the endosperm cells adjacent to the aleurone layer (sub-aleurone) are mitotically more active than the inner starchy endosperm cells and retain their mitotic activity until later during seed development (Sabelli and Larkins, 2009). Thus, a developmental gradient exists within a seed, since the cells in the sub-aleurone are younger than those found in deeper layers of the endosperm (starchy endosperm). The centripetal maturation of the endosperm is revealed in Figure 1A, showing a representative cross section comprising aleurone, sub-aleurone and first starchy endosperm cell layers of a maize seed at mid-developmental stage, when the PBs are already formed (Stage 2, Arcalis et al., 2010). Cells in the sub-aleurone are smaller and contain many electron-transparent vacuole-like structures and very few, small starch grains. Only three to four cell layers deeper, in the starchy endosperm, the morphology of the cells is completely different: they are much larger and are at least double the size of the sub-aleurone cells. Moreover, the number of vacuole-like structures in the starchy endosperm is significantly reduced and abundant, large starch grains are observed (Figure 1A). In addition, numerous small PBs of around 600 nm in diameter are present, as well as highly electron-dense oil bodies. Large and abundant ER strands, with ribosome-studded membranes are characteristic of these cells (Figure 1B and Supplementary Figure S1).

**FIGURE 2:**
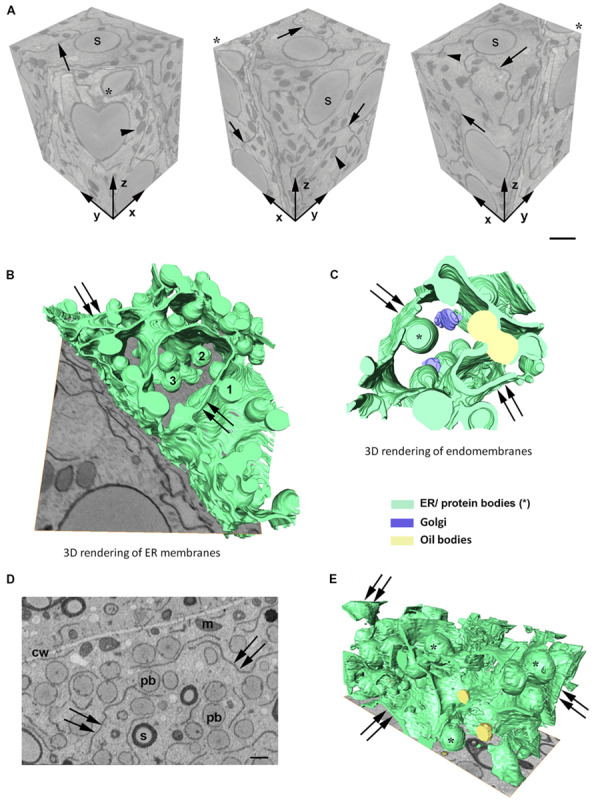
**(A)** Serial block face imaging of a starchy endosperm cell at mid-developmental stage (14 dap, Stage 2) and cut at 40-nm increments. Reconstructed volume, shown from different angles (asterisk marks the same corner at different positions). Note the high resolution in the *z* axis. Arrows mark the ER and arrowheads the protein bodies. Number of slices: 350, total volume: 2646 μm^3^, pixel size 20 × 20 nm. Scale bar, 1 μm. **(B–E)** 3D rendering of membranes in endosperm cells at different developmental stages. **(B,C)** Developmental stage 2 (14 dap). **(D,E)** Developmental stage 3 (21 dap). 3D rendering of ER membranes **(B)** and endomembranes **(C)** in a starchy endosperm cell. Massive ER sheets (double arrows) and abundant protein bodies (1, 2, 3) are budding from different *z*-positions **(B)**. Oil bodies bud from ER sheets (double arrows) neighboring the domains where PBs are formed **(C)**. **(D)** Representative z-stack image showing shorter ER strands (double arrow) and abundant protein bodies (>1 μm, pb). Cell wall (cw), mitochondria (m), starch (s). Scale bar, 1 μm. **(E)** 3D rendering of membranes. Smaller ER sheets (double arrow), protein bodies (^∗^).

In order to investigate the 3D organization of the ER and PBs in starchy endosperm cells in developing maize seeds, SBF-SEM was performed in samples corresponding to two different developmental stages: 14 daps (stage 2) and 21 daps (stage 3). A representative data set was selected for generating a 3D model. Figure 2A shows a reconstructed volume of a younger cell (stage 2) of around 2600 μm^3^ from different angles, with a resolution of 20 × 20 × 40 nm. The high lateral (*x*, *y*) and axial (*z*) resolution (slice thickness) enabled 3D analysis of specific ER strands from the block face (*xy*) that extended in the depth through the *z* plane, along 14 μm. It is also interesting to note the tangential disposition of the PBs to the ER strands (Figure 2A). Although these observations on the planes of the z-stack already give an impression of the 3D structure of the ER and the disposition of the PBs, it is only when those structures are rendered in a model that the architecture of the ER can be properly observed. Thus, the 3D rendering of the ER membranes (Figure 2B) shows that at mid-developmental stage, maize endosperm cells possess massive ER sheets, extending all through the cell, and that the presence of tubules is negligible. Additionally, it can also be observed that the PBs, as well as oil bodies, bud from different z-planes of the entire ER sheets. Moreover, according to these models, oil bodies appear randomly distributed in domains neighboring PBs (Figures 2C,E and Supplementary Video S1). In later developmental stages, when the zein synthesis reaches its peak, the model is also valid. At 21 dap, the ER-PB network has become much denser. While the number of oil bodies is not significantly higher, PBs are more abundant and larger (Figures 2D,E). It is interesting to note that in older seeds, the cisternae are still predominant but are not as large as in younger cells, probably because increasing portions of ER membrane are now forming part of the nascent PBs (Figures 2D,E).

## Discussion

The ER constitutes the entry point of the secretory pathway and plays a crucial role in the biosynthesis of diverse molecules within the cells. It is the organelle with the largest membrane surface area, consisting of a network of tubules and cisternae. Its morphology depends on the cell specialization and developmental stage (Stefano et al., 2014), and also other parts of the endomembrane system are subject to dynamic remodeling in response to physiological and developmental cues. As observed in Figure 1, the changes in the endomembrane system of maize endosperm cells are obvious and occur rapidly, since even adjacent cells show a distinct morphology. Thus, cells immediately under the sub-aleurone contain numerous vacuole-like structures and fewer PBs, whereas the neighboring starchy endosperm cells are characterized by the frequent presence of PBs and remarkably long ER strands. These changes are not exclusively found in maize endosperm, but are a general feature of cereal endosperm cells, in which the synthesis of seed storage proteins is accompanied by their deposition in storage organelles that are formed *de novo* (Arcalis et al., 2014). The apparent expansion of the ER that is accompanied by the appearance of the first PBs could be due to the increasing synthesis of zeins and might be needed to facilitate the formidable task for the synthetic machinery. Another example of ER expansion upon onset of high protein synthesis rates can be found in the B-cells of the mammalian immune system. Upon antigen stimulation, B-type lymphocytes can reach secretion rates of 3000 antibody molecules per second (King and Corley, 1989). In order to accommodate such an enormous protein load, the ER in these cells changes from rudimentary in a steady stage to a well-developed ER network containing the whole chaperon machinery needed for correct protein folding. Under the electron microscope, stimulated B-cells, as well as other highly differentiated secretory cells, like pancreatic exocrine cells, show a highly compacted ER ultrastructure, with rough ER cisternae densely studded with ribosomes (Barlowe, 2010; Kirk et al., 2010). Although maize endosperm cells do not reach the level of protein synthesis typically found in B-cells, parallels can be observed in the ER morphology, indicating that seed storage protein synthesis is enough to shift the balance in favor of sheets. There are different factors that control the rearrangement of the plant ER, including the actin cytoskeleton (Sparkes et al., 2009) and ER-shaping proteins like reticulons. Reticulons are found in areas of the ER with high curvature, e.g., in tubules (Shibata et al., 2008; Zurek et al., 2011), but they also play a role in the stabilization of the edge of ER cisternae (Tolley et al., 2008, 2010). Loss of function of ROOT HAIR DEFECTIVE 3 (RHD3), which participates in the shaping of the plant ER, is lethal, thus indicating that the control of the ER shape is important for the cell (Stefano and Brandizzi, 2018). In order to fully understand the mechanisms controlling ER morphology in endosperm cells, the expression patterns of these different plant ER shapers along endosperm maturation need to be determined and correlated with developmental studies at the submicron level.

It has been an open question whether PB formation and budding occurs preferably in ER areas with high curvature, such as tubules or the edge of the cisternae, or rather in flat areas of sheets. The anecdotic presence of ER tubules in developing maize endosperm cells, together with the fact that ribosomes are less frequently found on the surface of tubules (Shibata et al., 2006, 2010), indicates that a preference for PB formation from sheets is more likely. The use of 3D electron microscopy allowed us to conclude that PBs are formed within ER cisternae and that the entire surface of these cisternae is suitable for budding, without obvious ER domains being dedicated to the formation of PBs. Indeed, we could observe several oil bodies budding from the same ER cisternae in close proximity to PBs, indicating that no specific domains are defined in the ER of maize endosperm cells for the production of proteins or lipids.

Zeins are packed within the ER in spherical protein bodies with a final diameter of around 1 μm. Similar to other cereals like rice, wheat or barley, prolamins packed in PBs do not proceed through the secretory pathway in maize because they are transport incompatible. A goal in this study was to establish whether the majority of zein bodies remain in contact with the ER lumen or become terminal cytosolic organelles. Several previous studies have addressed this question using ectopically induced PBs. Llop-Tous et al. (2010) determined that mature Zera-induced PBs in *Nicotiana benthamiana* leaves remained connected with the ER. By co-expressing a soluble fluorescent protein in the ER (YFP-KDEL), they could observe that after photobleaching, YFP-KDEL diffused rapidly through the ER to reach the periphery of the bleached protein bodies. Similarly, Saberianfar et al. (2016) performed a series of photoconversion experiments on zein bodies also ectopically expressed in *N. benthamiana* leaves. Different to photobleaching, the recovery of fluorescence by newly synthesized protein is minimized when using photoconversion. The irreversible conversion of GFP into DsRed allowed the authors to track the photoconverted GFP within a PB or a group of PBs to neighboring or even distal PBs, providing evidence that PBs communicate with each other through the ER, and therefore the authors concluded that they remain attached to the ER (Saberianfar and Menassa, 2017). Based on their observations, the authors postulated a working model of PB formation and development. With our study, we provide information about the native ER-PB network at an ultrastructural level and we combine the high resolution of TEM with 3D modeling, allowing us to confirm that zein bodies are connected with the continuous ER network, not only in heterologous systems, but in the native system, i.e., the maize seed.

In conclusion, our study demonstrates that the development of 3D electron microscopy imaging techniques for ultrastructural volume reconstruction allows to address long-standing questions regarding the spatial relationship of membrane organelles and opens a new and exciting perspective on the endomembrane system of plant cells.

## Data Availability Statement

The original contributions presented in the study are included in the article/Supplementary Material, further inquiries can be directed to the corresponding author/s.

## Author Contributions

EA and ES contributed to the conception and design of the study and wrote the manuscript. EA and UH-D designed and carried out the electron microscopy and SBF-SEM experiments. EA and LZ analyzed the data and generated the 3D models. All authors contributed to manuscript revision, read, and approved the submitted version.

## Conflict of Interest

The authors declare that the research was conducted in the absence of any commercial or financial relationships that could be construed as a potential conflict of interest.

## Supplementary Material

FIGURE S1 Transmission Electron Microscopy. Developing endosperm cell at mid-developmental stage. Ribosomes decorate the membrane around the protein body (pb) and also the ER membrane (arrowheads). Protein storage vacuole (PSV), rough endoplasmic reticulum (rER), starch (s). Scale bar, 0.5 μm.

VIDEO S1 3D model of ER and protein bodies in young endosperm cells (stage 2).
