# Corrigendum: 3D Electron Microscopy Gives a Clue: Maize Zein Bodies Bud From Central Areas of ER Sheets

**DOI:** 10.3389/fpls.2020.01266

**Published:** 2020-08-18

**Authors:** Elsa Arcalis, Ulrike Hörmann-Dietrich, Lukas Zeh, Eva Stoger

**Affiliations:** Department of Applied Genetics and Cell Biology, University of Natural Resources and Life Sciences, Vienna, Vienna, Austria

**Keywords:** cereal endosperm, electron microscopy, endomembrane system, endoplasmic reticulum, maize, protein bodies, volume electron microscopy

## Missing Funding

In the original article, we neglected to acknowledge the HRSM project NANOBILD for infrastructure support. The corrected acknowledgment statement appears below:

The authors thank Dietmar Pum (Department of Nanobiotechnology, BOKU, Vienna) for technical advice and acknowledge the HRSM project NANOBILD for infrastructure support.

The authors apologize for this error and state that this does not change the scientific conclusions of the article in any way. The original article has been updated.

