# Supplementary figures and images for: 3D Electron Microscopy Gives a Clue: Maize Zein Bodies Bud From Central Areas of ER Sheets
Source: Front Plant Sci. 2020 Jun 11;11:809. doi: 10.3389/fpls.2020.00809 (PMC7301906; doi:10.3389/fpls.2020.00809)

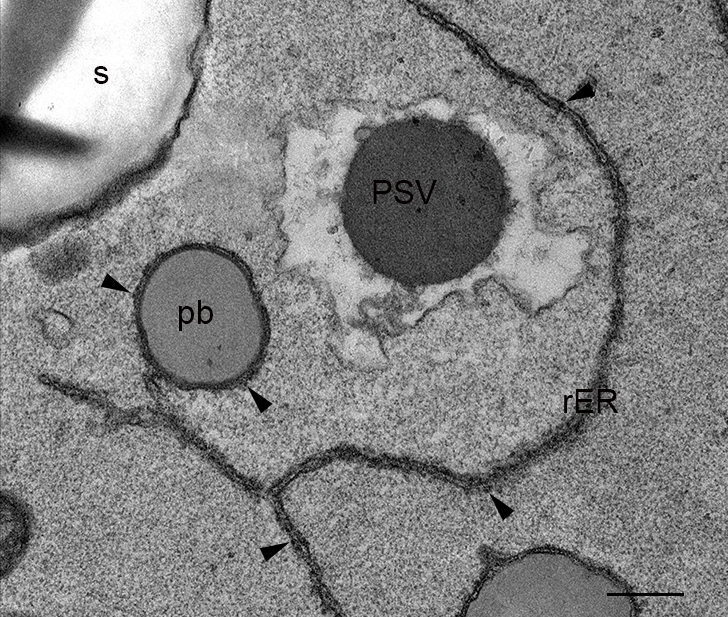

Supplement: FIGURE S1 — Transmission Electron Microscopy. Developing endosperm cell at mid-developmental stage. Ribosomes decorate the membrane around the protein body (pb) and also the ER membrane (arrowheads). Protein storage vacuole (PSV), rough endoplasmic reticulum (rER), starch (s). Scale bar, 0.5 μm. [file Image_1.TIF]
